# Insulin-Mimetic Activity of Herbal Extracts Identified with Large-Scale Total Internal Reflection Fluorescence Microscopy

**DOI:** 10.3390/nu16142182

**Published:** 2024-07-09

**Authors:** Cathrina Neuhauser, Bettina Schwarzinger, Clemens Schwarzinger, Michaela Feichtinger, Verena Stadlbauer, Verena Arnaut, Ivana Drotarova, Bernhard Blank-Landeshammer, Julian Weghuber

**Affiliations:** 1 Center of Excellence Food Technology and Nutrition, University of Applied Sciences Upper Austria, Stelzhamerstraße 23, 4600 Wels, Austria; cathrina.neuhauser@fh-wels.at (C.N.); bettina.schwarzinger@fh-wels.at (B.S.); michaela.feichtinger@fh-wels.at (M.F.); verena.stadlbauer@fh-wels.at (V.S.); verena.arnaut@fh-wels.at (V.A.); ivana.drotarova@fh-wels.at (I.D.); 2 FFoQSI GmbH-Austrian Competence Centre for Feed and Food Quality, Safety and Innovation, Technopark 1D, 3430 Tulln, Austria; bernhard.blank-landeshammer@fh-wels.at; 3 Institute for Chemical Technology of Organic Materials, Johannes Kepler University, 4040 Linz, Austria; clemens.schwarzinger@jku.at

**Keywords:** diabetes mellitus, insulin-mimetic compounds, GLUT4 translocation, total internal reflection fluorescence microscopy (TIRFM), modified hens egg test (Gluc-HET), *Saponaria officinalis*, *Pulmonaria officinalis*, Acacia catechu, Mentha spicata

## Abstract

Diabetes mellitus is a spreading global pandemic. Type 2 diabetes mellitus (T2DM) is the predominant form of diabetes, in which a reduction in blood glucose uptake is caused by impaired glucose transporter 4 (GLUT4) translocation to the plasma membrane in adipose and muscle cells. Antihyperglycemic drugs play a pivotal role in ameliorating diabetes symptoms but often are associated with side effects. Hence, novel antidiabetic compounds and nutraceutical candidates are urgently needed. Phytogenic therapy can support the prevention and amelioration of impaired glucose homeostasis. Using total internal reflection fluorescence microscopy (TIRFM), 772 plant extracts of an open-access plant extract library were screened for their GLUT4 translocation activation potential, resulting in 9% positive hits. Based on commercial interest and TIRFM assay-based GLUT4 translocation activation, some of these extracts were selected, and their blood glucose-reducing effects in ovo were investigated using a modified hen’s egg test (Gluc-HET). To identify the active plant part, some of the available candidate plants were prepared in-house from blossoms, leaves, stems, or roots and tested. *Acacia catechu* (catechu), *Pulmonaria officinalis* (lungwort), *Mentha spicata* (spearmint), and *Saponaria officinalis* (common soapwort) revealed their potentials as antidiabetic nutraceuticals, with common soapwort containing GLUT4 translocation-activating saponarin.

## 1. Introduction

Hyperglycemia is characteristic of diabetes mellitus, which is a metabolic disorder. Excessive blood glucose can cause polyuria, polydipsia, infection susceptibility, impaired vision, ketoacidosis, and a risk of coma [1]. Diabetes mellitus can be classified into different types, and type 2 diabetes mellitus (T2DM) is the predominant form [2]. T2DM patients have impaired insulin response, insulin secretory defects, and increased glucose production in the liver [3,4]. Mostly obese patients > 40 years are affected by T2DM [1]. Genetic and metabolic factors, including an unhealthy diet, lack of physical activity, and smoking, influence T2DM disease onset and progression [2]. Symptoms of T2DM are commonly ameliorated with the antihyperglycemic agent, metformin, which belongs to the class of biguanides [5], along with insulin therapy [6] and physical exercise [7]. Other medications used are sulfonylureas, thiazolidinediones, glucagon-like peptide-1 (GLP-1) receptor agonists, and dipeptidyl peptidase-4 (DPP-4) inhibitors [5]. However, these types of drugs are associated with side effects. Specifically, increased body weight and hypoglycemia are side effects of insulin glargine treatment [8], and sulfonylureas and rosiglitazone, a thiazolidinedione drug, are associated with a potentially higher risk of cardiovascular events [9,10]. Furthermore, a case study of a 78-year-old patient reported metformin-promoted cholestatic and hepatocellular liver injury [11].

It is estimated that in 2045, more than 780 million people worldwide will suffer from diabetes, and more than USD 1000 billion will be spent on medication and therapy in the United States [12].

In untreated T2DM patients, insulin-resistant cells are a key priority of investigation. In healthy individuals, glucose uptake into insulin-sensitive adipose and muscle cells is stimulated by insulin and insulin-independent pathways [13,14,15]. GLUT4s are present intracellularly in basal cells, with only a few being displayed on the cell surface [13,16]. GLUT4 translocation can be stimulated by physical exercise and energy depletion in an insulin-independent manner via AMP-activated protein kinase (AMPK) signaling in skeletal muscle, heart, liver, or adipose tissue [14,15]. GLUT4-mediated glucose trafficking is activated via the p38 mitogen-activated protein kinase (MAPK) downstream molecule after muscle contraction [17,18]. For insulin-dependent signaling, insulin binding to the insulin receptor displayed on the cell surface stimulates receptor autophosphorylation and recruitment of insulin receptor substrate (IRS) proteins, as well as activation of phosphatidylinositol 3 kinase (PI3K), formation of phosphatidylinositol 3,4,5-trisphosphate (PIP_3_), activation of protein kinase C-alpha (PKCα), and activation of protein kinase b (Akt). This enzymatic reaction promotes the translocation of GLUT4 from intracellular GLUT4 storage vesicles (GSVs) to the plasma membrane, facilitating blood glucose transport from the extracellular space to the intracellular space, thus maintaining glucose homeostasis [13]. In T2DM, this mechanism is impaired [13,19].

The prevention and amelioration of T2DM symptoms can be supported by phytogenic therapy. Because plants produce secondary metabolites as a defense mechanism against environmental stressors (e.g., insects and pathogens) [20,21], they are a source of nutraceuticals, and knowledge about their beneficial effects exists from traditional medicine. Numerous plants have been reported to reduce blood glucose levels, including bitter melon, ginseng, onion, garlic, aloe vera [22], and goat’s rue. Galegine, which is hypoglycemic but toxic, was isolated from goat’s rue, and the study of galegine resulted in the development of metformin [23]. Plants consist of many phytochemical and bioactive compounds, with only a few of them causing the desired effect in a specific application. The yield and composition of the metabolite profile depend on the extraction method [24,25,26,27], plant part (e.g., leaves, roots, or blossoms) [24,28,29], geographic effects [30], and seasonal influences [31,32,33]. Phenolic compounds, including flavonoids (e.g., kaempferol, quercetin, and naringenin), alkaloids (e.g., berberine), terpenoids [such as saponins (e.g., ginsenosides and oleanolic acid)], sterols, carotenoids, essential oils, coumarins, and tannins, have been reported to influence blood glucose levels [34].

To identify new GLUT4 translocation activator extracts, the present study investigated plant extracts from an open-access plant extract library (PECKISH) [35]. GLUT4 translocation was visualized using TIRFM, a highly sensitive scanning tool, which is a proven technique for screening approaches near the cell membrane, e.g., visualizing GLUT4 plasma membrane insertion by an increase in GLUT4-green fluorescent protein (GFP) signal in HeLa cells [36,37,38,39]. After a primary screen, the most effective insulin-mimetic plant extracts were selected, including those of potential commercial interest, and their blood glucose-lowering effects were measured in ovo (Gluc-HET) [40,41] to support their application as nutraceuticals or food supplements. Extracts prepared from *Acacia catechu* (catechu), *Saponaria officinalis* (common soapwort), *Pulmonaria officinalis* (lungwort), and *Mentha spicata* (spearmint) showed the highest efficacy. As the plant part used for extraction influences activity [24,28,29], some extracts were prepared in-house from plant leaves, blossoms, stems, or roots to investigate GLUT4 membrane insertion. Moreover, the blood glucose-reducing potentials of common soapwort blossom plus stem extract, common soapwort leaf extract, lungwort blossom extract, lungwort leaf extract, and spearmint leaf extract were investigated in ovo.

## 2. Materials and Methods

### 2.1. Reagents

Potassium dihydrogen phosphate [KH_2_PO_4_ (5 mM)], HEPES (20 mM), magnesium sulfate [MgSO_4_ (1 mM)], calcium chloride [CaCl_2_ (1 mM)], sodium chloride [NaCl (136 mM)], potassium chloride [KCl (4.7 mM)], sodium hydroxide (NaOH), human insulin, Roswell Park Memorial Institute (RPMI) 1640 cell culture medium, dimethyl sulfoxide (DMSO), and Hank’s balanced salt solution (HBSS) were purchased from Sigma-Aldrich (Schnelldorf, Germany). Phosphate-buffered saline (PBS) was purchased from PAN-Biotech (Aidenbach, Germany). Trypsin/EDTA, penicillin/streptomycin, Paneticin G 418, and fetal bovine serum (FBS) were purchased from M&B Stricker (Bernried, Germany). The PECKISH plant extract library containing > 2000 water-soluble extracts was obtained from Prof. Frank Döring (Christian-Albrechts University, Kiel, Germany) [35]. NovoRapid insulin (Novo Nordisk, Bagsvaerd, Denmark) was a gift from Daniel Weghuber (Paracelsus Medical University, Salzburg, Austria). Saponarin was purchased from Extrasynthese (Genay, France), and menisdaurin was purchased from Oskar Tropitzsch GmbH (Marktredwitz, Germany).

### 2.2. In-House Extract Preparation

Fresh plants were collected locally in Upper Austria or purchased from a regional vendor who obtains plants from farmers in Upper Austria or Styria. Fresh plant material was dried at 50 °C overnight and ground with a coffee grinder (De’Longhi-Kenwood GmbH, Wiener Neudorf, Austria). Cold and hot extracts of the plants were prepared. Five grams of the powder was suspended in water, vortexed, sonicated for 30 min, shaken for 2 h, and centrifuged. The supernatant and residues were collected, and they were diluted in 50 mL of water and aliquoted. The same method was applied for hot extraction, with the following alterations: (1) the dried plant material was dissolved in hot water (70 °C); (2) the samples were shaken for 10 min; and (3) the samples were diluted in 70 °C hot water before being aliquoted.

### 2.3. Total Internal Reflection Fluorescence Microscopy (TIRFM)

TIRFM measurements were performed as previously reported [36,37], with some minor alterations. Briefly, 40,000 HeLa-GLUT4-myc-GFP cells per well were seeded into 96-well imaging plates (MoBiTec, Goettingen, Germany), grown overnight, washed twice with HBSS, and starved for 3 h with HBSS. The cells were stimulated with PECKISH (1:1000 and 1:10,000) or in-house extracts (1:1000 and 1:10,000), human insulin (100 nM), menisdaurin (600 nM), and saponarin (prediluted in DMSO; tested at 840 nM) dissolved in Krebs Ringer Phosphate HEPES (KRPH) buffer (pH = 7.4). Images before and after stimulation were recorded at 10 min time intervals using TIRFM. TIRFM was performed using an epifluorescence microscope (Nikon Eclipse Ti2, Tokyo, Japan) with an automated Perfect Focus System. A 60× CFI Plan-Apochromat objective was used, and the scanning of multiple stage positions was supported by a motorized x-y-stage (CMR-STG-MHIX2, Märzhäuser, Germany). Emission diode lasers (Toptica Photonics, Munich, Germany) were used for the excitation of GFP at 488 nm, and the appropriately filtered fluorescence signal was recorded by an sCMOS camera (Andor Zyla 4.2) (Oxford Instruments, Abingdon, UK).

### 2.4. Modified Hens Egg Test (Gluc-HET)

The Gluc-HET test was conducted as previously reported [40,41]. After 11 days of incubation at 38 °C, 40–60% humidity, and constant turning in an egg incubator (HEKA Brutgeräte, Rietberg, Germany), the air bladders of fertilized chicken eggs (Lohmann classic brown chicken) were marked by candling. Using a pointed pair of tweezers, the eggshell was pecked, and 300 µL of the plant extract (dilution 1:16) or the NovoRapid insulin analog (3 U/mL) diluted in water was injected with a syringe into the air bladder. After an incubation period of 60 to 120 min and removal of the eggshell above the air bladder, the eggshell membrane was wetted with PBS and removed. The chorioallantoic membrane was cut, and a suitable blood vessel was placed on a pH strip. The blood vessel was swabbed and opened, and the blood was collected with a pipette and transferred to the measuring strip of a blood glucose meter (Accu-Chek Performa, Roche Diabetes Care GmbH, Mannheim, Germany).

### 2.5. High-Performance Liquid Chromatography (HPLC) Analysis

Analysis of common soapwort leaf, common soapwort stem + blossom, lungwort leaf, lungwort blossom, PECKISH common soapwort, and PECKISH lungwort extracts was performed using reversed-phase chromatography with a Thermo Scientific Dionex Ultimate 3000 comprising an LPG-3400SD pump with a built-in degasser, a WPS-3000 U(T)SL cooled autosampler, a temperature-controlled column compartment, and Chromeleon software (7.2 software package) as described previously [42]. Analytes were separated on an Accucore C18 column (150 mm × 3.0 mm inner diameter, 2.6 µm particle size; Thermo Fisher Scientific), with a column temperature of 40 °C, an injection volume of 1 µL, and ultraviolet (UV) wavelength detection at 260 nm. The analytes were separated by gradient elution with mobile phase A (0.1% formic acid in water) and mobile phase B (0.1% formic acid in acetonitrile) at a flow rate of 0.5 mL/min. The elution was started with 95% A and 5% B. The elution gradient was performed as follows: B was increased to 20% after 5 min of equilibration time; B was increased to 40% after 12 min; B was increased to 60% after 15 min; B was increased to 80% after 17 min for 3 min; and at 20 min, B was reduced to 5% after 25 min. High-resolution mass spectra were obtained using a linear trap quadrupole (LTQ) Orbitrap Velos from Thermo Fisher Scientific equipped with an atmospheric pressure chemical ionization (APCI) source operated in both positive and negative ionization mode at a resolution of 30,000. Based on mass determination using liquid chromatography–mass spectrometry (LC–MS), the saponarin and mensidaurin polyphenols were preidentified and then quantified with an HPLC diode array detector (DAD) against known standards at concentrations ranging from 1 to 1000 mg/L.

### 2.6. Data Analysis

TIRFM images were recorded by NIS-Elements AR software (version 5.02.01 64-bit, Nikon, Tokyo, Japan). For comparison of fluorescence intensity values after different treatments and time intervals, regions of interest (ROIs) of all cells mapped on an image were selected in the Spotty framework (https://bioinformatics.fh-hagenberg.at/site/fileadmin/user_upload/img_upload/projects/spotty.html; accessed on 19 June 2024). The mean intensity values were corrected to background values and KRPH signals. The fluorescence intensity of GLUT4-GFP 20 min after stimulation with the extracts is used for further analysis. Additional images were taken at 10 and 30 min poststimulation, and these data are provided in the Supplementary Materials (Supplementary Figures S1–S3 and S5).

Blood glucose levels after treatment with the plant extracts were normalized and corrected to those of untreated eggs and water controls.

The data were controlled for a Gaussian distribution with the Shapiro-Wilk test and analyzed with the Kruskal-Wallis test, with a 95% confidence interval, using GraphPad Prism (version 6.07, GraphPad Software Inc., San Diego, CA, USA). *p* values ≤ 0.05 were considered significant (* *p* ≤ 0.05, ** *p* ≤ 0.01, *** *p* ≤ 0.001, and **** *p* ≤ 0.0001).

Figures were prepared using GraphPad Prism version 6.07, heatmaps using GraphPad Prism version 9.0.0, and Corel Draw (version 2019, Corel Corporation, Ottawa, ON, Canada).

The graphic abstract was created in BioRender (Science Suite Inc., Toronto, ON, Canada).

For generating the taxonomic tree, R statistical software (version 3.6.2, the R project for statistical computing, Vienna, Austria), with the taxize package for retrieval of taxonomic data and metacoder for visualization, was used [43,44].

## 3. Results

### 3.1. Investigation of GLUT4 Translocation Activity of 772 Plant Extracts in HeLa-GLUT4-myc-GFP Cells

**Figure 1:**
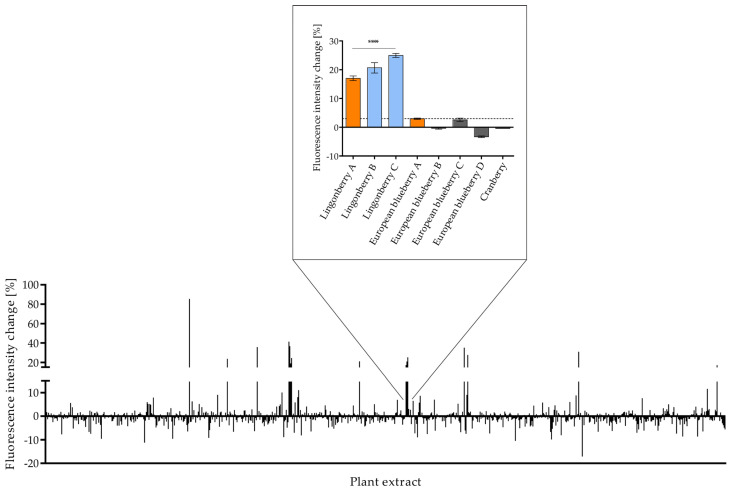
Quantification of the GLUT4-myc-GFP signal in HeLa cells after incubation with 772 plant extracts of the open-access plant extract library (PECKISH) [35]. Cells were seeded in 96-well imaging plates, grown overnight, washed, starved in HBSS for 3 h, and stimulated with extracts (1:10,000). TIRFM images were taken before and after stimulation. The time point at 20 min after stimulation is shown. The data are shown as the mean ± SEM (n > 40). The mean intensity values were corrected to the background and KRPH signal. Representative lingonberry, European blueberry, and cranberry extract activity is shown in the magnification. A threshold of 3% was defined for positive hits (dashed line) [36]. **** *p* < 0.0001 indicates statistically significant differences compared with the KRPH control. The blue and orange bars represent positive hits. The orange-colored state extracts were investigated in more detail in ovo (refer to Section 3.4).

**Figure 2:**
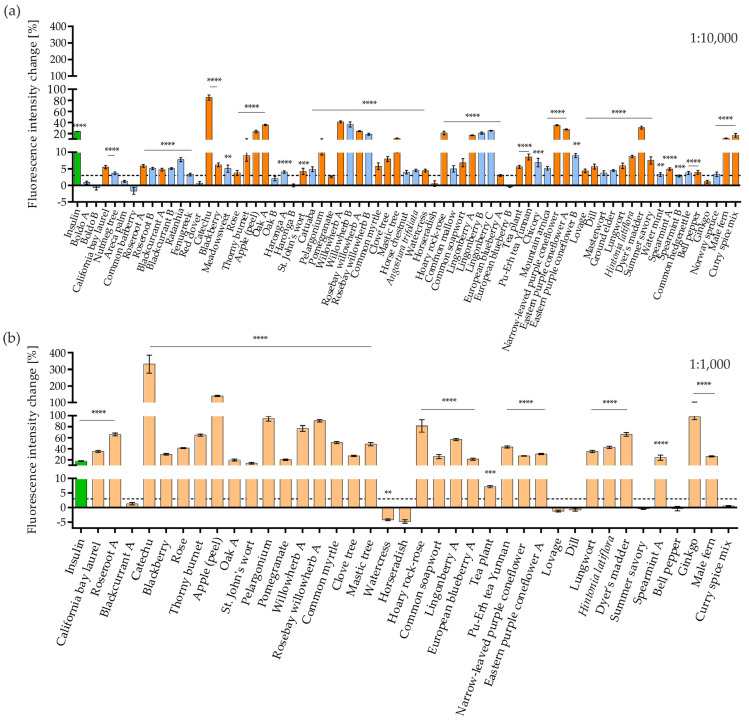
The effects of GLUT4 translocation in HeLa GLUT4-myc-GFP cells were quantified 20 min after stimulation with PECKISH plant extracts [35] or 100 nM insulin. Cells were seeded in 96-well imaging plates, grown overnight, washed, and starved in HBSS for 3 h, after which they were imaged with TIRFM and stimulated with the extracts. A threshold of 3% was defined for positive hits (dashed line) [36]. Extracts with a positive effect (**a**) from the initial screen at a dilution of 1:10,000 are indicated. (**b**) Signal of PECKISH extracts recently obtained from the same plant source at a dilution of 1:1000. Data are shown as the mean ± SEM (n > 41). The mean intensity values were corrected to the background and KRPH signal. **** *p* < 0.0001, *** *p* < 0.001, and ** *p* < 0.01 indicate statistically significant differences compared with the KRPH control.

To investigate GLUT4 translocation after stimulating live cells with plant extracts, we previously established and optimized a high-content screening TIRFM assay [36,37,39]. This technology was used to screen 772 aqueous plant extracts from the open-access PECKISH library [35] in HeLa cells stably expressing a GLUT4-myc-GFP construct (Figure 1). Although CHO-K1 hIR/GLUT4-myc-GFP and 3T3-L1 GKUT4-GFP cells have been previously used for screening, HeLa cells were used in the present study because they are easy to manipulate and express the human insulin receptor, allowing insulin sensitivity (Figure 2a,b) [37]. Insulin was applied at a concentration of 100 nM as a positive control throughout the present experiments.

After cells were starved using HBSS, they were stimulated with the extracts (1:10,000), and the change in GLUT4-GFP fluorescence signal intensity in the evanescent field was determined. As in a previous study, a threshold of greater than 3% fluorescence signal intensity increase was considered a positive effect [36]. The primary screen revealed 70 GLUT4 translocation active extracts from 57 different plants. These primary hit extracts were retested at least twice. As an example, the activity of eight of these extracts, namely, lingonberry (*Vaccinium vitis-idea*), European blueberry (*Vaccinium myrtillus*), and cranberry (*Vaccinium macrocarpon*), are shown in Figure 1.

### 3.2. Validation of Extracts Stimulating GLUT4 Translocation with Newly Prepared Extracts from the Same Source

Because of the low throughput and high extraction volumes required to investigate the blood glucose-lowering potential of GLUT4 translocation-active plant extracts in ovo (refer to Section 3.4), 38 PECKISH extracts were selected based on commercial interest and GLUT4 translocation efficacy (>5%). The extracts were generated from fresh preparations and validated for their effects on the GLUT4-GFP signal.

Figure 2a shows the imaging screen at the 20 min time point using a 1:10,000 dilution of the extracts. Figure 2b shows the GLUT4 translocation activity achieved with the preselected freshly prepared extracts at a 1:1000 dilution. A comparative analysis revealed that not all the plants in which GLUT4 translocation was stimulated in the initial screen were active according to the reproduced extracts. Freshly prepared extracts of horseradish (*Armoracia rusticana*), watercress (*Nasturtium officinale*), lovage (*Levisticum officinale*), bell pepper (genus *Capsicum*, species not defined in PECKISH), dill (*Anethum graveolens*), summer savory (*Satureja hortensis*), and curry spice mix were inactive at all time points. However, repetitively active extracts showed increased GLUT4 translocation activity at higher concentrations (1:1000 dilution) (Figure 2b).

Catechu, apple (*Pyrus malus*; prepared from peel), oak A (*Quercus robur*), willowherb A and B (genus *Epilobium*, species not defined in PECKISH), rosebay willowherb A (*Chamaenerion angustifolium*), hoary rock-rose (*Cistus incanus)*, lingonberry, narrow-leaved purple coneflower (*Echinacea angustifolia*), eastern purple coneflower A (*Echinacea purpurae*), and dyer’s madder (*Rubia tinctorum*) extracts were most effective in the initial screen, with an increase in GLUT4-GFP intensity greater than 20% (Figure 2a). These findings were confirmed by testing freshly prepared extracts. The extracts of roseroot A (genus *Rhodiola*, species not defined in PECKISH), catechu, thorny burnet (*Poterium spinosum*), apple (peel), pelargonium (genus *Pelargonium*, species not defined in PECKISH), willowherb A, rosebay willowherb A, common myrtle (*Myrtus communis*), mastic tree (*Pistacia lentiscus*), hoary rock-rose, lingonberry A, dyer’s madder, and ginkgo (*Ginkgo biloba*) showed an intensity increase greater than 45%. The effects of blackberry (*Rubus fruticosus*), oak A, common soapwort, eastern purple coneflower A, lungwort, and spearmint A were also prominent. Blackcurrant A (*Ribes nigrum*) showed activity after 10 min but not after 20 or 30 min (refer to Supplementary Figure S2).

### 3.3. Determination of Active Plant Parts Using in-House Prepared Extracts from Leaves, Blossoms, Stems, or Roots from Regionally Available and GLUT4 Translocation-Active PECKISH Plants

As plant parts differ in their phytochemical profile [24,28,29], determining which plant parts or combinations of different parts are responsible for GLUT4 translocation activity is key for further use. Therefore, 19 plant extracts with active GLUT4 translocation in the initial screen (refer to Figure 2a) were selected for detailed analysis. The extracts were prepared in-house from the blossoms, leaves, stems, or roots of these plant species and tested for GLUT4 translocation activity. The plants were selected based on their regional availability and included red clover (*Trifolium pratense*), blackberry, rose (*Rosa*), St. John’s wort (*Hypericum perforatum*), watercress, horseradish, *Malva* (only genus, species not defined), common soapwort, lingonberry, European blueberry, chicory (*Cichorium intybus*), lovage, dill, ground elder (*Aegopodium podagraria*), lungwort, summer savory, water mint (*Mentha aquatica*), spearmint, and green bell pepper (*Capsicum annuum*). For these plants, the location of growth, time of harvest, and extraction method (hot or cold) are known.

**Figure 3:**
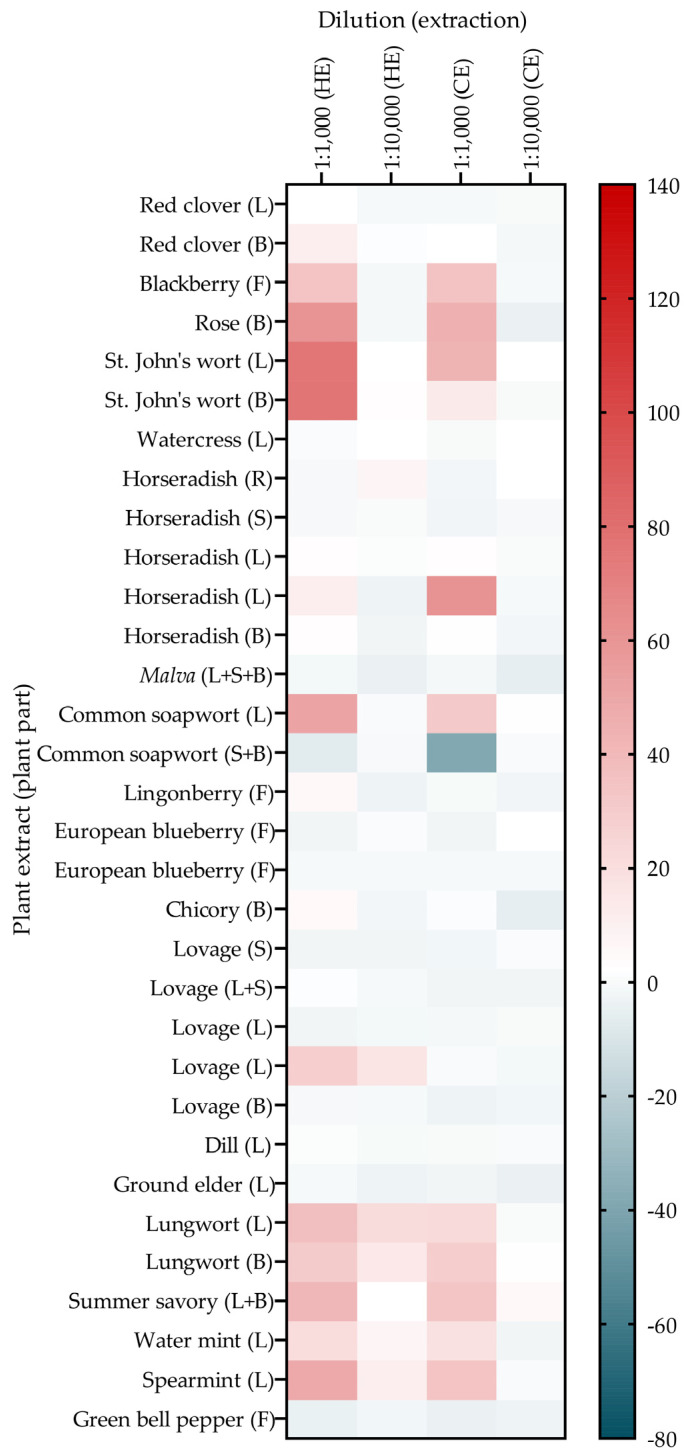
Quantified GLUT4 translocation in HeLa GLUT4-myc-GFP cells (%) of in-house prepared extracts at 20 min after stimulation. Plant extracts were prepared from the blossoms (B), fruits (F), leaves (L), roots (R), or stems (S) with hot (HE) or cold extraction (CE). Cells were seeded in 96-well imaging plates, grown overnight, washed, starved in HBSS for 3 h, imaged with TIRFM, and stimulated with the extracts at 1:1000 and 1:10,000 dilution. Mean intensity values were corrected to the background and KRPH signal. The mean of data (n > 37) was used to develop the color code depicted in the legend. Red indicates a GLUT4-myc-GFP signal increase, while blue indicates a GLUT4-myc-GFP signal decrease.

The results confirmed that GLUT4 translocation activity differed depending on the plant part used for extraction (Figure 3). The common soapwort leaf extract increased the fluorescence intensity of GLUT4-GFP, whereas the combined common soapwort stem and blossom extract had no positive effect on GLUT4 translocation. The red clover blossom extract from hot extraction increased the fluorescence signal, but the red clover leaf extract did not increase the signal. The blackberry (fruit), rose (blossom), St. John’s wort (leaf and blossom), horseradish (both leaf extracts), lingonberry (fruit), lovage (one of two leaf extracts), lungwort (leaf and blossom), summer savory (leaf and blossom combination), water mint (leaf), and spearmint (leaf) extracts induced GLUT4 translocation. No effects were detected for the horseradish (root, stem, and blossom), *Malva* (leaf, stem, and blossom combination), European blueberry (fruit), chicory (blossom), lovage (stem, leaf, and blossom), dill (leaf), ground elder (leaf), or green bell pepper (fruit) extracts. High concentrations (1:1000) of insulin-mimetic extracts resulted either in increased GLUT4-GFP signals, unchanged, or reduced signals that might be caused by cell detachment. Compared with cold extraction, hot extraction of the plant parts was slightly more efficient and led to an increased effect.

### 3.4. Investigation of Changes in Blood Glucose Levels in Chicken Embryos of GLUT4 Translocation-Active Plant Extracts (In Ovo)

**Figure 4:**
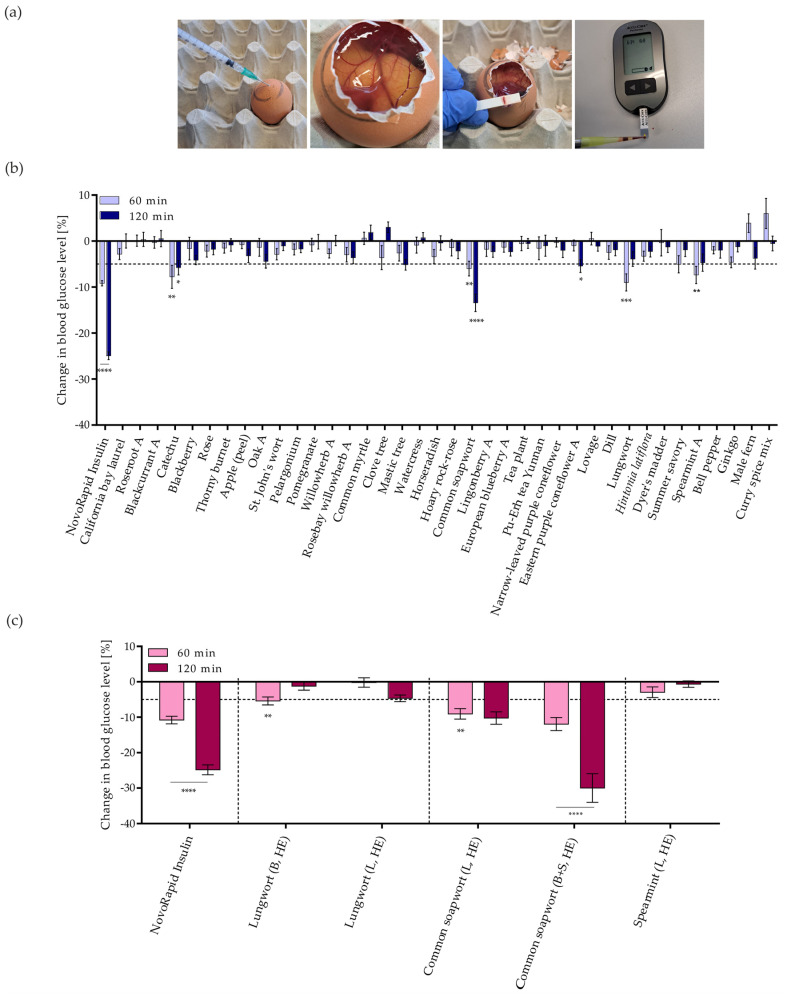
Blood glucose changes in ovo using the modified hen’s egg test (Gluc-HET) after stimulation with plant extracts. NovoRapid insulin at 3 U/mL was used as the positive control. (**a**) After incubation for 11 days, the air bladder above the chorioallantoic membrane was marked, and the plant extract was injected. After 60 or 120 min of incubation, the egg was opened, the membrane was removed, blood was collected from the main vessel, and the blood glucose level of the chicken embryo was measured using a blood glucose meter. (**b**) Blood glucose level changes after stimulation with extracts of the open-access plant extract library (PECKISH) [35] at a 1:16 dilution. (**c**) Blood glucose reduction for in-house lungwort, common soapwort, and spearmint extracts at dilutions of 1:16. Abbreviations: B = blossom, L = leaf, B + S = blossom + stem, and HE = hot-extracted. Blood glucose levels after treatment with the plant extracts were normalized to those of untreated eggs and water controls. A threshold of -5% was defined for positive hits (dashed line) [36]. Data are shown as the mean ± SEM (n > 7). **** *p* < 0.0001, *** *p* < 0.001, ** *p* < 0.01, and * *p* < 0.05 indicate statistically significant differences compared with the water control.

To further investigate the plant extracts that induce GLUT4 translocation, the effects of these extracts on blood glucose levels in chicken embryos were determined in vivo, using the previously described Gluc-HET method [36,38,40,41,45]. Because the throughput of this method is low, only 38 PECKISH extracts with active GLUT4 translocation were selected based on commercial interest and the increase in the intensity of the cellular GLUT4-GFP signal (refer to Figure 2b). After applying the extracts (diluted 1:16) onto the chorioallantoic membrane (CAM) of a fertilized egg, the eggs were incubated for 60 and 120 min before the eggshell and eggshell membrane were removed, and the blood was collected and used to measure the blood glucose concentration (Figure 4a). Similar to a previous study, the present study considered a decrease in blood glucose of less than −5% to indicate a positive effect [36].

Figure 4b shows the effect of the selected PECKISH extracts on egg blood glucose levels. The catechu and common soapwort extracts exceeded the threshold of −5% after 60 and 120 min of incubation, showing significance. Lungwort and spearmint resulted in significant reductions in blood glucose levels after 60 min of incubation. The blood glucose levels after applying blackberry (after 120 min), oak (after 120 min), and ginkgo (after 60 min) tended to decrease and almost exceeded the threshold but showed no significance. Summer savory exceeded the threshold after 60 min, and mastic tree and eastern purple coneflower after 120 min of incubation. The primary hit extracts were retested at least twice.

To identify the plant part responsible for common soapwort, lungwort, and spearmint activity, the in-house prepared common soapwort, lungwort, and spearmint extracts (hot extracted) were tested. Figure 4c shows the effects of the lungwort (leaf vs. blossom), common soapwort (leaf vs. stem + blossom), and spearmint (leaf) extracts on blood glucose levels. The hot-prepared lungwort blossom extract was effective after 60 min of incubation but exhibited a loss of activity after 120 min, whereas blood glucose levels after incubation with the lungwort leaf extract were reduced more after 120 min than after 60 min. The common soapwort extracts prepared from leaves and a combination of stem and blossoms efficiently reduced blood glucose levels after 60 and 120 min of incubation, and they achieved blood glucose reductions greater than 25%, whereas the spearmint leaf extract did not show a significant blood glucose reduction.

### 3.5. Identification of Phytochemicals in Lungwort and Common Soapwort Extracts

**Figure 5:**
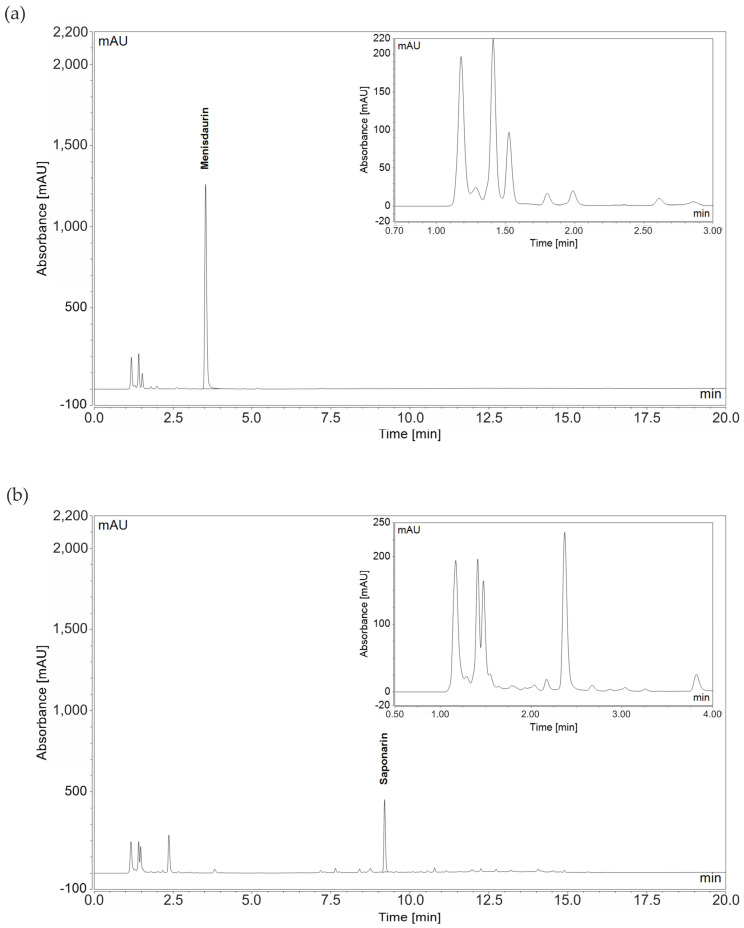
HPLC-diode array detector (DAD) chromatograms of in-house prepared (**a**) lungwort leaf extract (hot extracted) and (**b**) common soapwort leaf extract (hot extracted) at 260 nm. Menisdaurin (**a**) and saponarin (**b**) were identified. The concentrations of menisdaurin (retention time 3.5 min) and saponarin (retention time 9.2 min) are shown in Table 1. The unassigned peaks between retention times of 0.7 and 3 min (**a**) and between 0.5 and 4 min (**b**) are magnified.

Because of the antihyperglycemic potential of the common soapwort and lungwort extracts, the main components of both in-house prepared extracts were identified using HPLC high-resolution MS (as described in Section 2.5), and their concentrations were determined using HPLC-DAD by external calibration using standards. In the lungwort extract, the main peak at a retention time of 3.5 min consisted of only mensidaurin (MH^+^_observed_ = 314.1231 vs. MH^+^_calculated_ = 314.1234), and the smaller peaks were not assigned. In the common soapwort extract, the main compound was saponarin (MH^+^_observed_ = 595.1663 vs. MH^+^_calculated_ = 595.1659), and the smaller peaks were not assigned (Figure 5). The concentrations of the pure compounds in the different plant parts after both hot and cold extraction are shown in Table 1. Additionally, saponarin was identified in the PECKISH common soapwort extract, but the PECKISH lungwort extract lacked menisdaurin. Additional HPLC-diode array detector chromatograms of the in-house prepared hot-extracted blossom lungwort extracts and blossom + stem common soapwort extracts as well as of the PECKISH lungwort and common soapwort extracts are included in the Supplementary Materials (refer to Supplementary Figure S4).

**Figure 6:**
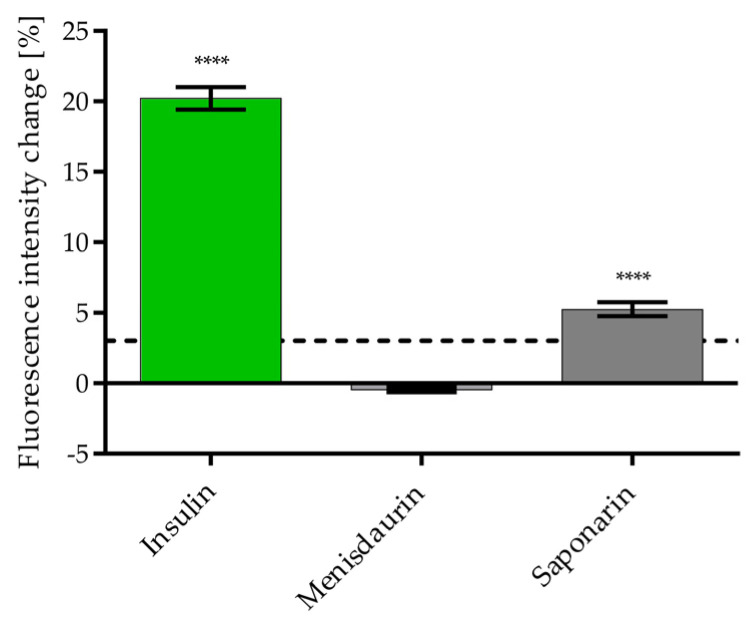
Quantification of GLUT4 translocation in HeLa GLUT4-myc-GFP cells 20 min after stimulation with 100 nM insulin, 600 nM menisdaurin, and 840 nM saponarin. Cells were seeded in 96-well imaging plates, grown overnight, washed, starved in HBSS for 3 h, imaged with TIRFM, and stimulated with the compounds. A threshold of 3% was defined for a positive signal (dashed line) [36]. Data are shown as the mean ± SEM (n > 115). The mean intensity values were corrected to the background and KRPH signal. **** *p* < 0.0001 indicates a statistically significant difference compared with the KRPH control.

The potential of the pure compounds to induce GLUT4 translocation was determined by TIRFM. To compare the results, the concentrations of the pure compounds used were adjusted to the tested concentrations on the screen (compared with a 1:1000 dilution, Figure 3). Saponarin induced GLUT4 translocation at 840 nM and menisdaurin (at 600 nM) had no effect on plasma membrane GLUT4 levels (Figure 6). Cell viability was not affected by saponarin and menisdaurin at the tested concentrations (refer to Figure S5c in the Supplementary Materials).

## 4. Discussion

In the present study, 772 plant extracts from an open-access plant extract library (PECKISH) [35] were tested for their GLUT4 translocation-inducing potential, with approximately 9% of all screened extracts representing positive hits. These high hit rates were comparable to that of a previous study conducted by our group [36]. The same or similar phytochemicals and phytochemical combinations occur in different plants, explaining the high hit rates. The 772 tested extracts belonged to 423 different plants, 57 of which exhibited active GLUT4 translocation. To attenuate negative effects that may arise from plant toxicity or highly autofluorescent extracts, the primary screen was performed at a dilution of 1:10,000. However, because of experimental throughput limitations, only 5% of all screened extracts that were positive could be further investigated in ovo (Gluc-HET). Before in ovo testing, the selected extracts were freshly prepared and retested for GLUT4 translocation activity at a higher concentration (1:1000). In this validation step, not all plant extracts showed effects compared to the initial screen. However, most of the extracts showed concentration-dependent activities. These differences in performance may be due to concentration effects (higher concentrations resulting in more toxicity in some cases), autofluorescent effects, or other effects arising from more concentrated phytochemicals. In a previous study, we discussed the effects of extracts with strong autofluorescence and toxicity. Minimal to medium autofluorescence normally has no significant influence on the detection signal, as in TIRFM, only the plasma membrane of cells is visualized and the autofluorescence of the cytoplasm is unresolved [37]. Cell detachment from the glass surface of the 96-well plate and hence loss of fluorescence signal might be caused by high levels of certain phytochemicals at higher extract concentrations. Some phytochemicals may have toxic effects or cause pH changes in wells, leading to cell detachment. Additionally, studies have revealed that depending on the plant part used for extract preparation, differences in phytochemical profiles arise [24,28,29]. Hence, in-house prepared extracts of known plant parts, which were cold and hot extracted, were used to validate some of the initial hits from the PECKISH screen. The present study identified differences in GLUT4 translocation efficiency between the in-house-made and PECKISH extracts. Specifically, the leaf extract of common soapwort enhanced the GLUT4-GFP signal, whereas the combined stem and blossom extract had no positive effect. Treatment with the combined stem and blossom common soapwort extract resulted in cell detachment, suggesting either toxicity of the extract or soap-like action of certain phytochemicals. In addition, GLUT4 translocation in other plant species, such as lungwort, was induced by the leaf and blossom. Also, the spearmint leaf extract strongly induced GLUT4 translocation. The presence of the GLUT4-GFP signal increased in both horseradish leaf and in one of two lovage leaf extracts, whereas GLUT4 translocation did not change in any of the other tested plant parts, suggesting that the PECKISH horseradish/lovage extracts used in the initial screen may contain a combination of several plant parts, with the leaf part being major in the initial screen and possibly minor in the freshly prepared extracts. Further investigation of the top 5% most promising hits resulting from the initial screen using the Gluc-HET assay revealed significant blood glucose reductions exceeding −5% for the catechu, spearmint, common soapwort, and lungwort extracts. Additionally, the mastic tree and eastern-purple coneflower extracts showed blood glucose reductions > 5% (after 120 min of incubation); however, these results were not further investigated because the effect after 60 min of incubation was not relevant. The in-house-prepared common soapwort, lungwort, and spearmint extracts were tested in ovo, with spearmint leaf extract inducing no significant blood glucose reduction. There was strong blood glucose reduction after treatment with the common soapwort leaf extract and the common soapwort stem + blossom extract. Although the effect was lower, there was an efficient blood glucose reduction after treatment with the lungwort blossom extract and a reductive trend for the lungwort leaf extract. HPLC analysis of these extracts revealed the presence of saponarin in common soapwort and of menisdaurin in lungwort. Saponarin, also present in the PECKISH extract, is known to induce AMPK-dependent GLUT4 expression [46], and the present study confirmed the GLUT4 translocation activity of pure saponarin. The lack of GLUT4 translocation activity of menisdaurin and its absence in the PECKISH lungwort extract gives an additional strong hint on the activity of other compounds.

**Figure 7:**
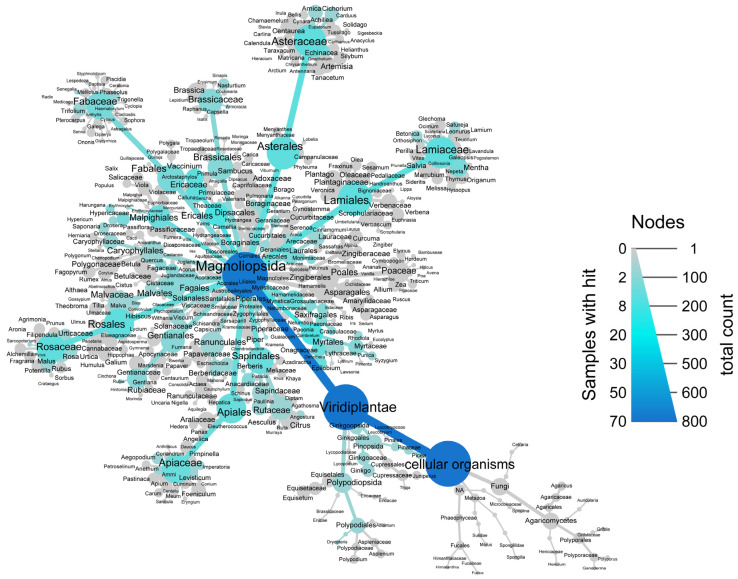
Taxonomic tree indicating all tested extracts from the plant extract library (PECKISH) [35]. The size of the nodes represents the number of samples tested for GLUT4 translocation activity, and the color intensity represents the number of positive hits. Nine percent of the 772 tested plant extracts induced GLUT4 translocation in HeLa GLUT4-myc-GFP cells and were considered positive hits.

To correlate the plants with GLUT4 translocation-inducing activity from the initial screen with their plant order or family, the taxonomy of these plants was explored (Figure 7). Most of the 423 investigated plants belonged to the class Magnoliopsida in the clade Viridiplantae [47,48], the minority of which were fungal extracts. The plants with GLUT4 translocation-inducing ability were distributed across many plant orders. In total, 27–45% of the plants belonging to Laurales, Saxifragales, Myrtales, Malvales, or Ericales exhibited active GLUT4 translocation. However, upon a closer look at other plant families with GLUT4 translocation-inducing activity, these numbers decreased, with only 7–21% of the plants belonging to Ranunculales, Fabales, Rosales, Sapindales, Brassicales, Caryophyllales, Asterales, Apiales, Gentianales, and Lamiales having positive hits. The hypothesis that GLUT4 translocation activity is correlated with plant order or plant family was not validated in the present study. However, the present study tested only 423 plants, which is a small portion of plants.

Because numerous phytochemicals, including polyphenols, alkaloids, and terpenoids, induce GLUT4 translocation [49], phytochemical clustering is rational. The concentration of GLUT4 translocation-inducing phytochemicals and their presence, depending on the plant part tested [24,28,29], are dependent on various parameters, such as seasonal influences [31,32,33], geographical influences [30,32], extraction method efficiency [24,25,26,27], and compound steric configuration [50,51]. GLUT4 level-influencing phytochemicals, such as quercetin [52,53], caffeic acid [54], kaempferol [55], rutin [56], chlorogenic acid [57], catechin [58], gallic acid [59], apigenin, baicalein, luteolin [60], ursolic acid [61], or isorhamnetin [53], are frequently present in different parts of various plants, representing potential candidates for GLUT4 translocation activity in the screened extracts.

An investigation of the plants that most effectively reduced blood glucose levels in ovo revealed that lungwort (*Pulmonaria officinalis*) can contain rutin, hesperidin, chlorogenic acid, myricetin, hyperoside, acacetin, gallic acid, naringenin [62,63], and numerous other phytochemicals. Lungwort is distributed across Europe and Asia [64], and it is used in traditional medicine as an anti-inflammatory agent and diuretic agent, as well as to treat cough [65,66]. The plant has been studied for its antioxidative and anti-neurodegenerative potential [67], and the aerial parts of the plant are considered in the National Health Products Ingredients Database of Canada for homeopathic use [68]. Some plant components are undesirable, such as pyrrolizidine alkaloids, which have been detected in many herbal tea formulations, including lungwort [69]. Hence, *Pulmonaria officinalis* is found on the negative list of the Austrian Food Code [70]. However, according to a previous study, pyrrolizidine alkaloids were not present in *Pulmonaria officinalis* leaves but in roots [71]. Dried aerial parts, dietary supplements, teas, and cosmetics derived from lungwort are commercially available [72,73].

Common soapwort (*Saponaria officinalis*), the second most effective extract in ovo, is native to Central Europe and is distributed in Western Asia, Northern Europe, the United States of America, and South America [74]. Common soapwort roots have been used as a soap substitute because the plant contains saponins, which possess soap-like properties, and their toxicity plays a role in plant defense against pathogens [21,75,76,77]. Additionally, potentially toxic type 1 ribosome-inactivating proteins (RIPs), such as saporins, are found in the aerial and underground parts of common soapwort [78,79,80]. Common soapwort is used for different purposes in traditional medicine. In Italy, the leaves are used as skin repellents, sanitizers, and diuretics, and they are also used to treat liver diseases [79,81,82]. The common soapwort plant has been studied for its antimicrobial, anticancer, hepatoprotective, anti-insecticidal, and antioxidant effects [79]. The phytochemicals that can be found in common soapwort include oleanolic acid [83] and saponarin [77].

Catechu (*Acacia catechu)* efficiently reduced blood glucose levels in ovo. Different parts of the catechu plant can contain phytochemicals such as acacetin, chrysin, diosmetin, myricetin, kaempferol, isorhamnetin, naringenin, taxifolin, quercetin, rutin, gallic acid, catechin, chlorogenic acid, epicatechin, caffeic acid, coumaric acid, umbelliferone, and ellagic acid [84,85,86]. Catechu is used in traditional medicine to treat gastrointestinal problems, leprosy, and skin diseases, and it is found in eastern and central Africa, as well as in Asian countries, including China, India, and Nepal [85]. Catechu has been reported to have anticancer [85,87], antiviral [85,88], antimicrobial [85,89], anti-inflammatory [85,90], immunomodulatory [85,91], antidiabetic, and antihyperglycemic properties [85,92,93,94,95]. Apart from traditional medicine, gum exudates from the plant are used as emulsifiers, adhesives, food, textiles, cosmetic stabilizers, and demulcents [85,96,97].

Also, the PECKISH spearmint (*Mentha spicata*) extract reduced blood glucose levels in ovo. Spearmint leaves can contain naringenin, epicatechin, catechin, apigenin, myricetin, caffeoylquinic acids, luteolin, and rutin [98]. The plant is commonly used as a tea or flavoring agent and has high commercial value. In traditional medicine, it has a broad field of use, including the treatment of respiratory issues and diabetes [99]. In vivo studies describe the antidiabetic effect of spearmint leaves [99,100], supporting the outcome of the GLUT4 translocation activity shown in this study.

## 5. Conclusions

In conclusion, the present study identified insulin-mimetic plants using a TIRFM-based screen and verified the blood glucose reduction effects of the extracts in ovo. The present findings indicate that plants, including common soapwort, lungwort, spearmint, and catechu, have potential as antidiabetic nutraceuticals. HPLC analysis revealed the presence of saponarin in common soapwort, and the TIRFM results indicate the induction of GLUT4 translocation by saponarin. Further, 9% of the GLUT4 translocation active extracts discovered in the initial TIRFM-based screen may have the potential to reduce hyperglycemia. Additional in vivo studies are needed to confirm the present results.

## Figures and Tables

**Table 1:** Concentrations of menisdaurin in in-house prepared lungwort and concentrations of saponarin in in-house prepared common soapwort were identified using HPLC–DAD and were quantified based on known standards. Abbreviations: B = blossom; L = leaf; B + S = blossom + stem; CE = cold extracted; HE = hot-extracted; and n.q. = not quantified.

Extract Name	Concentration of Compound	
	Menisdaurin [mg/mL]	Saponarin [mg/mL]
Lungwort (B, CE)	1.3638	n.q.
Lungwort (B, HE)	0.2172	n.q.
Lungwort (L, CE)	0.2958	n.q.
Lungwort (L, HE)	0.985	n.q.
Common soapwort (L, CE)	n.q.	1.0264
Common soapwort (L, HE)	n.q.	0.4682
Common soapwort (B+S, CE)	n.q.	1.1202
Common soapwort (B+S, HE)	n.q.	1.0664

## Data Availability

The data presented in this study are available on request from the corresponding author. All source data of the graphs are provided in a Supplementary Data File.

## Supplementary Materials

The following supporting information can be downloaded at https://www.mdpi.com/article/10.3390/nu16142182/s1, Figure S1: Quantified GLUT4 translocation in HeLa GLUT4-myc-GFP cells (a) 10 min and (b) 30 min after stimulation with 70 plant extracts collected in the open-access plant extract library (PECKISH); Figure S2: Quantified GLUT4 translocation in HeLa GLUT4-myc-GFP cells (a) 10 min and (b) 30 min after stimulation with 38 plant extracts collected in the open-access plant extract library (PECKISH); Figure S3: Quantified GLUT4 translocation in HeLa GLUT4-myc-GFP cells (%) (a) 10 min and (b) 30 min after stimulation with in-house prepared extracts; Figure S4: HPLC-diode array detector (DAD) chromatograms comparing (a) hot-extracted in-house lungwort blossom (pink line) or leaf (black line) extract with the PECKISH lungwort (blue line) extract and (b) the hot-extracted in-house common soapwort blossom + stem (black line) or leaf (pink line) extract with the PECKISH common soapwort (blue line) extract at 260 nm; Figure S5: Quantified GLUT4 translocation in HeLa GLUT4-myc-GFP cells (a) 10 min and (b) 30 min after stimulation with 100 nM insulin, 600 nM menisdaurin, and 840 nM saponarin.

## Abbreviations

AMPK: AMP-activated protein kinase; CAM: chorioallantoic membrane; DAD: diode array detector; DPP-4: dipeptidyl peptidase-4; GFP: green fluorescent protein; GLP-1: glucagon-like peptide-1; Gluc-HET: modified hen’s egg test for blood glucose analysis; GLUT4: glucose transporter 4; GSV: GLUT4 storage vesicle; KRPH: Krebs Ringer Phosphate HEPES; MAPK: mitogen-activated protein kinase; PECKISH: Plant Extract Collection Kiel in Schleswig-Holstein; T2DM: type 2 diabetes mellitus; TIRFM: total internal reflection fluorescence microscopy.
