# Supplementary material for: Insulin-Mimetic Activity of Herbal Extracts Identified with Large-Scale Total Internal Reflection Fluorescence Microscopy
Source: Nutrients. 2024 Jul 9;16(14):2182. doi: 10.3390/nu16142182 (PMC11280383; doi:10.3390/nu16142182)
Supplement: Supplementary file 1 [file nutrients-16-02182-s001.zip › Nehauser_et al_supplementary information_revised.pdf]

Supplementary Materials:

(a)

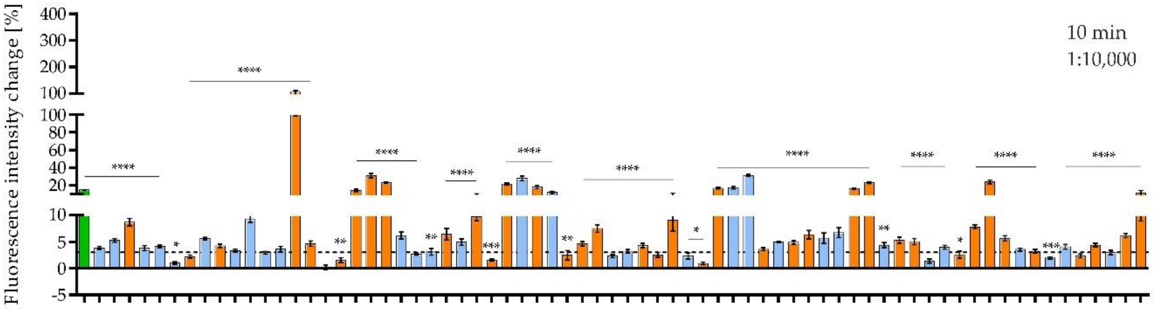

(b)

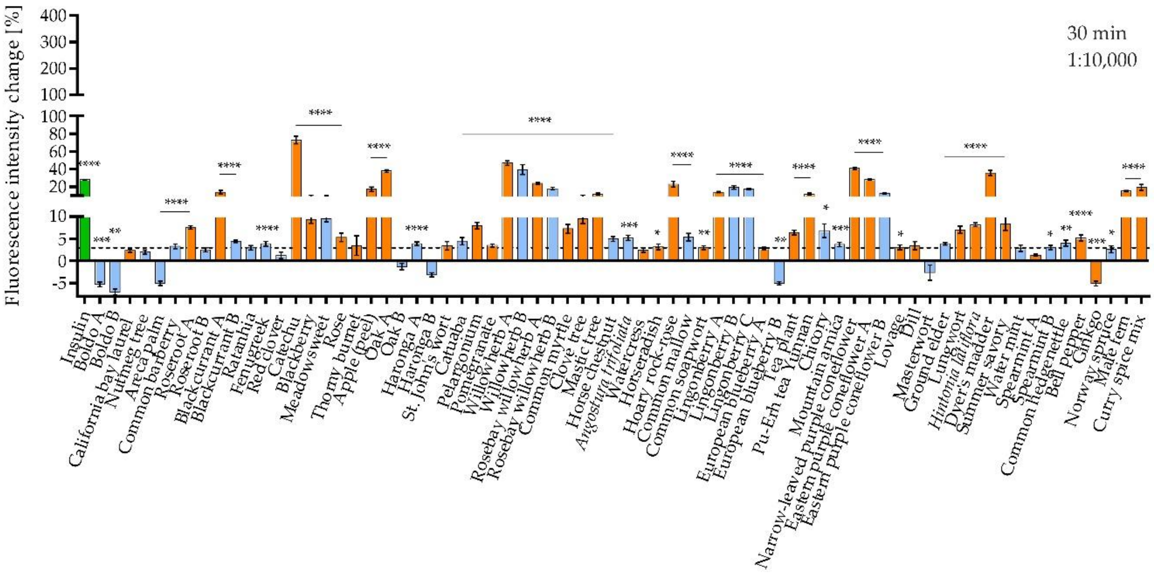

Supplementary Figure S1. Quantified GLUT4 translocation in HeLa GLUT4-myc-GFP cells, (a) 10 min, (b) 30 min after stimulation with 70 plant extracts collected in the open access plant extract library (PECKISH) or 100 nM insulin. Cells were seeded in 96-well imaging plates, grown overnight, washed, and starved in HBSS for 3 h, imaged with TIRFM and stimulated with the extracts. A threshold of 3% was defined for positive hits (dashed line). Indicated are extracts with a positive effect from the initial screen, dilution: 1:10,000. Blue and orange-colored bars represent the positive hits. The orange-colored state the extracts that were investigated in more detail in ovo. Data are shown as the mean +/- SEM (n > 41). Mean intensity values were corrected to background and KRPH signal. **** $p < 0.0001$, *** $p < 0.001$, ** $p < 0.01$ and * $p < 0.05$ indicate statistically significant differences from the KRPH control.

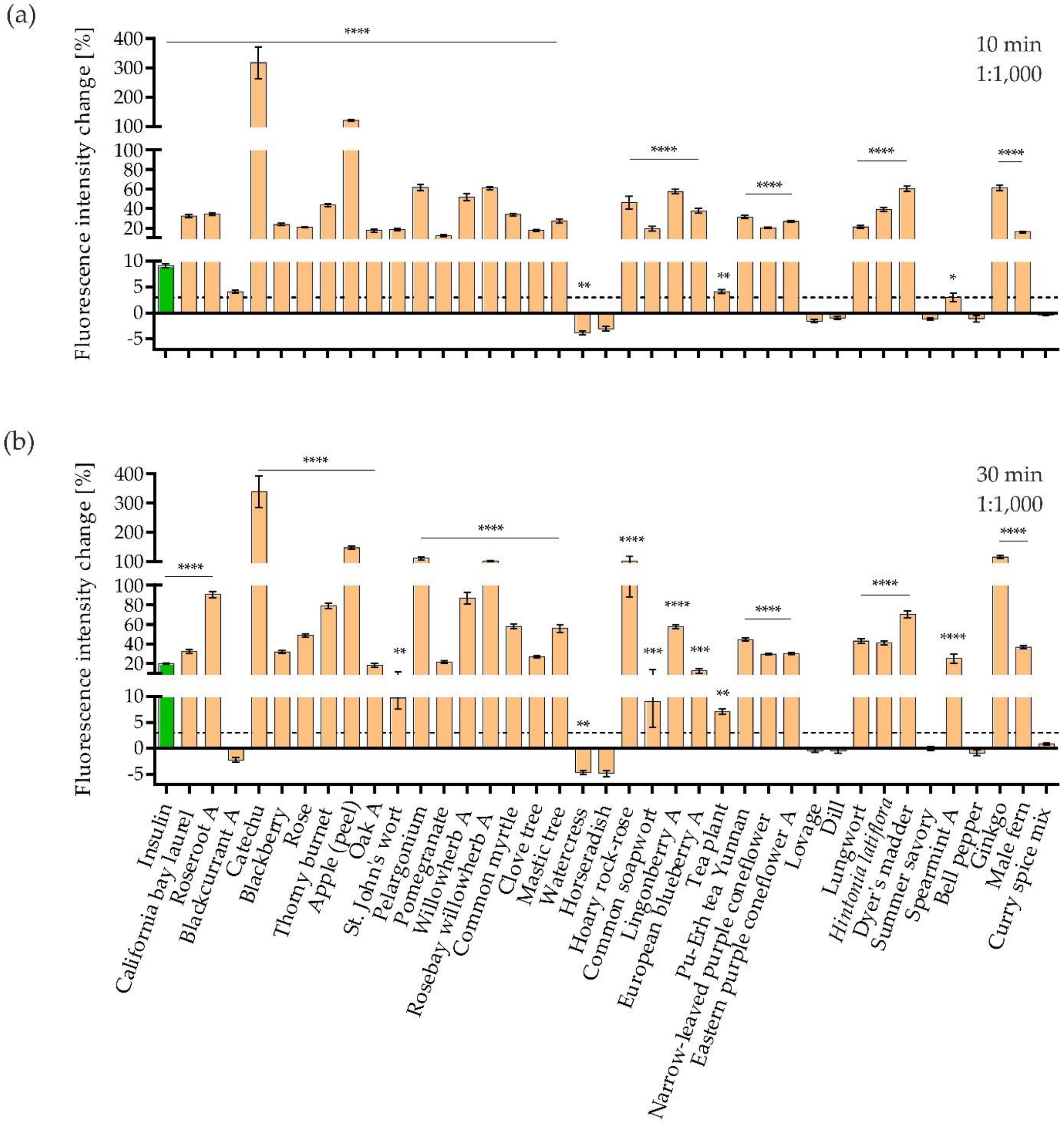

Supplementary Figure S2. Quantified GLUT4 translocation in HeLa GLUT4-myc-GFP cells, (a) 10 min, (b) 30 min after stimulation with 38 plant extracts collected in the open access plant extract library (PECKISH) or 100 nM insulin. Cells were seeded in 96-well imaging plates, grown overnight, washed, and starved in HBSS for 3 h, imaged with TIRFM and stimulated with the extracts. A threshold of 3% was defined for positive hits (dashed line). Indicated is the signal of recently prepared PECKISH extracts, dilution 1:1,000. Data are shown as the mean +/- SEM ($n > 41$). Mean intensity values were corrected to background and KRPH signal. **** $p < 0.0001$, *** $p < 0.001$, ** $p < 0.01$ and * $p < 0.05$ indicate statistically significant differences from the KRPH control.

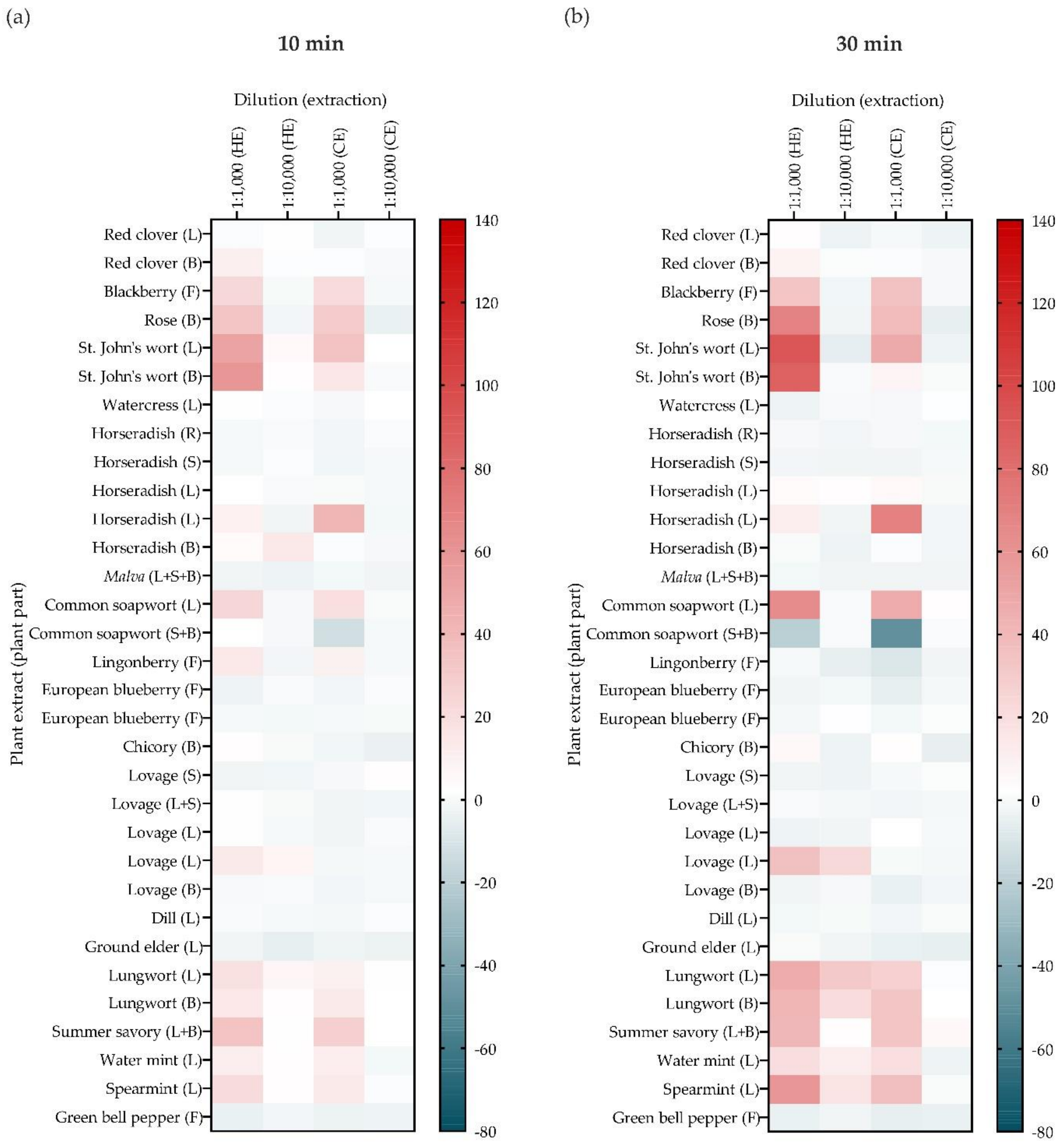

Supplementary Figure S3. Quantified GLUT4 translocation in HeLa GLUT4-myc-GFP cells (%), (a) 10 min, (b) 30 min after stimulation with in-house prepared extracts. Plant extracts were prepared from blossom (B), fruit (F), leaves (L), root (R) or stem (S) with hot (HE) or cold extraction (CE). Cells were seeded in 96-well imaging plates, grown overnight, washed, and starved in HBSS for 3 h, imaged with TIRFM and stimulated with the extracts at 1:1,000 and 1:10,000. Mean intensity values were corrected to background and KRPH signal. The mean of data (n > 37) was used to develop a color code depicted in the legend. Red indicates a GLUT4-myc-GFP signal increase, blue a signal decrease.

(a)

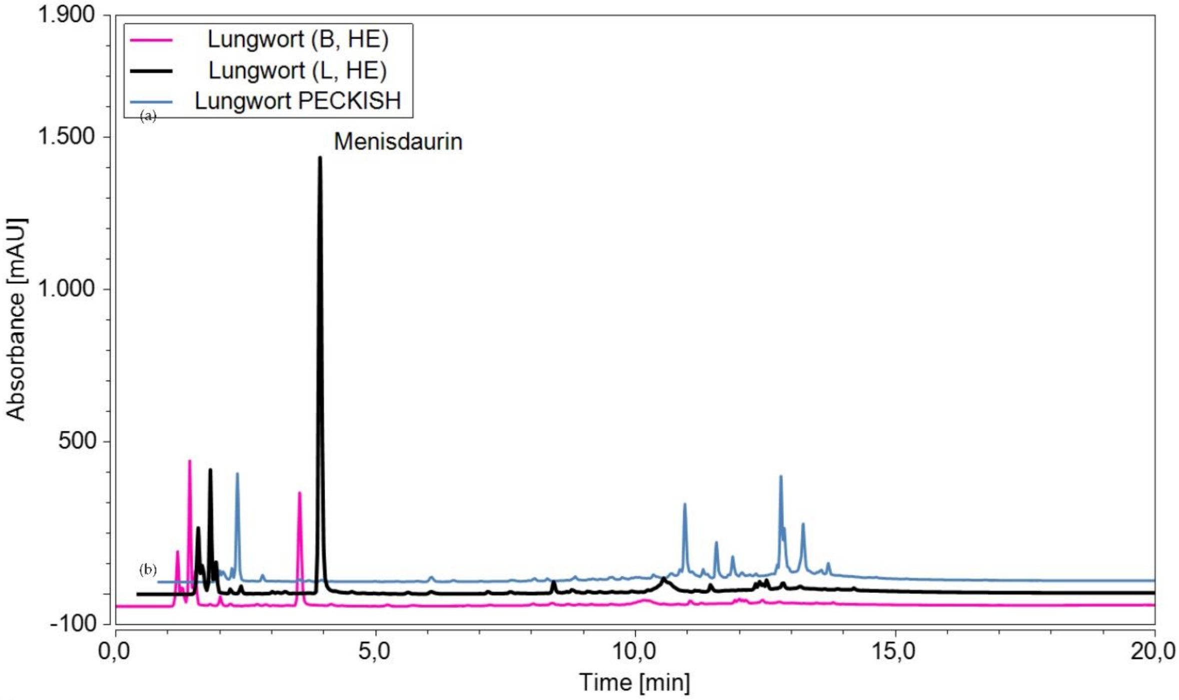

(b)

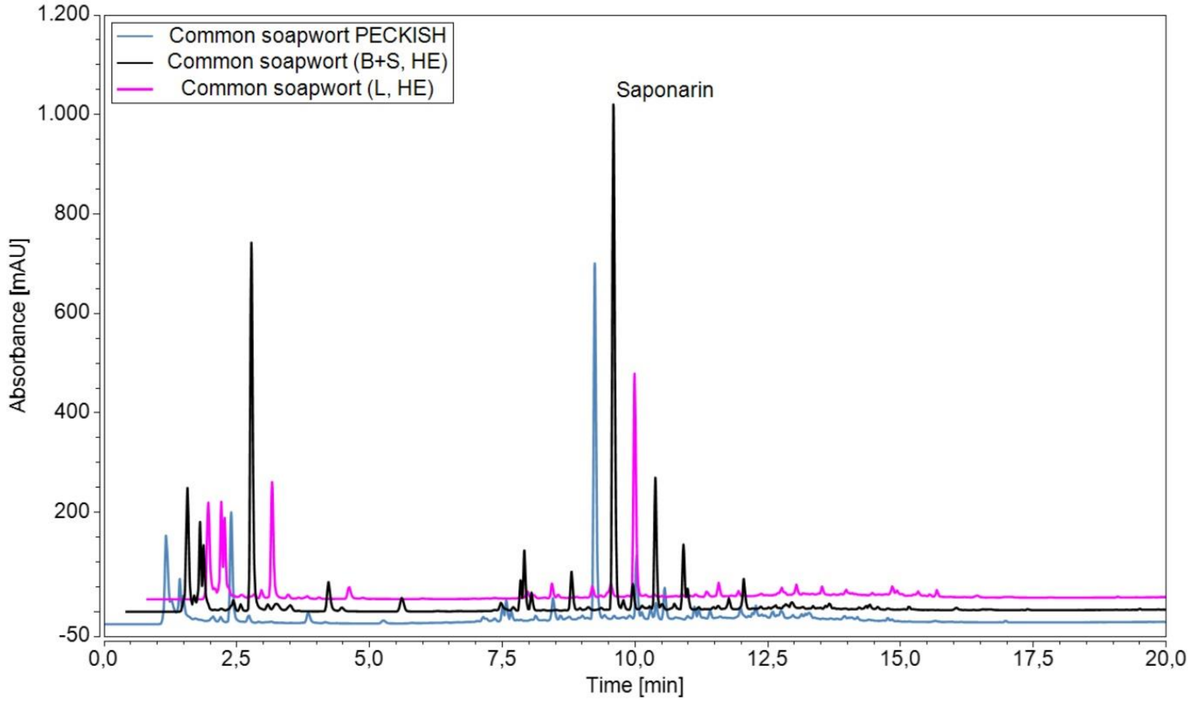

Supplementary Figure S4. HPLC-diode array detector (DAD) chromatograms comparing (a) hot extracted in-house lungwort blossom (pink line) or leaf (black line) extract with the PECKISH lungwort (blue line) extract and (b) the hot extracted in-house common soapwort blossom + stem (black line) or leaf (pink line) extract with the PECKISH common soapwort (blue line) extract at 260 nm. (a) Menisdaurin (retention time 3.5 min) was identified in in-house prepared lungwort extracts, but not in the PECKISH extract. (b) Saponarin (retention time 9.2 min) was identified in PECKISH and in in-house prepared common soapwort extracts. Abbreviations: B = blossom, L = leaf, B+S = blossom + stem, and HE = hot extracted.

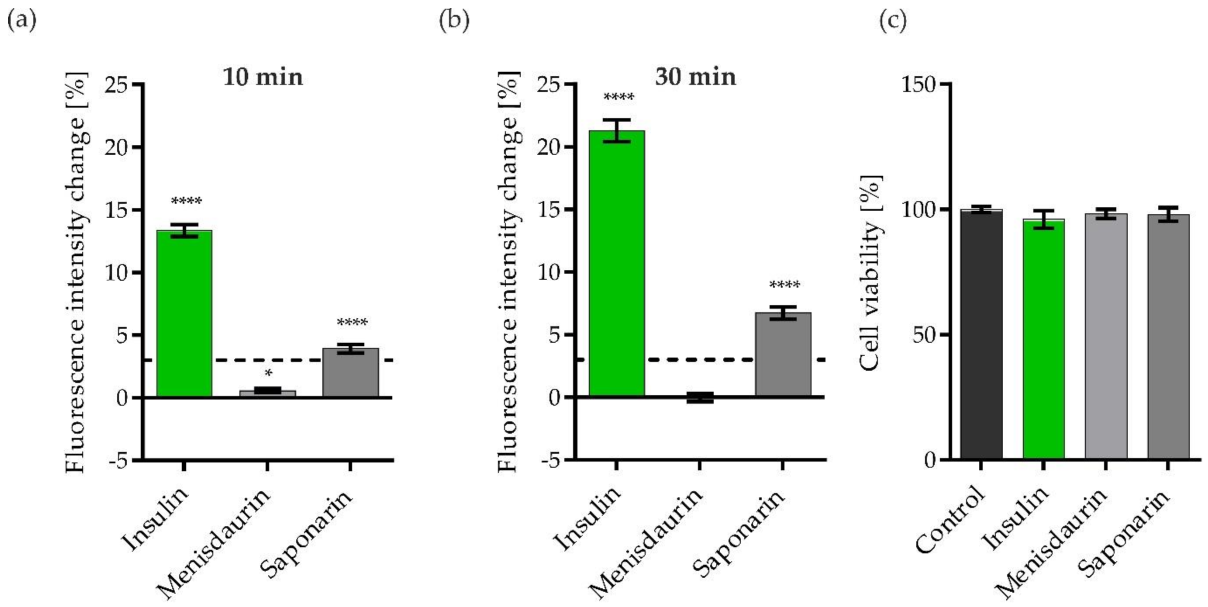

Supplementary Figure S5. Quantified GLUT4 translocation in HeLa GLUT4-myc-GFP cells, (a) 10 min, (b) 30 min after stimulation with 100 nM insulin, 600 nM menisdaurin and 840 nM saponarin. Cells were seeded in 96-well imaging plates, grown overnight, washed, and starved in HBSS for 3 h, imaged with TIRFM and stimulated with the extracts. A threshold of 3% was defined for positive signal (dashed line). Data are shown as the mean +/- SEM (n > 115). Mean intensity values were corrected to background and KRPH signal. **** $p < 0.0001$ and * $p < 0.05$ indicates statistically significant differences from the KRPH control. (c) Cell viability after treatment with 100 nM insulin, 600 nM menisdaurin, and 840 nM saponarin. No significant influence on cell viability of the tested substances was observed. Cells were seeded in 96-well plates, grown overnight, starved for 3 h, treated with insulin, menisdaurin and saponarin for 30 min, before incubation with Resazurin (1:20 dilution) for 1.5 h, and detection of fluorescent signal with an exication wavelength at 544 nm and emission wavelength at 590 nm. Data are shown as the mean +/- SEM (n > 5).
